# A Bone-Targeting Enoxacin Delivery System to Eradicate Staphylococcus Aureus-Related Implantation Infections and Bone Loss

**DOI:** 10.3389/fbioe.2021.749910

**Published:** 2021-11-16

**Authors:** Cong Yao, Meisong Zhu, Xiuguo Han, Qiang Xu, Min Dai, Tao Nie, Xuqiang Liu

**Affiliations:** ^1^ Department of Orthopedics, The First Affiliated Hospital of Nanchang University, Artificial Joints Engineering and Technology Research Center of Jiangxi Province, Nanchang, China; ^2^ Department of Orthopaedics, Xinhua Hospital Affiliated to Shanghai Jiaotong University School of Medicine, Shanghai, China

**Keywords:** implant infection, enoxacin, aspartic acid octapeptide, mesoporous silica nanoparticles, bone-targeting

## Abstract

Post-operative infections in orthopaedic implants are severe complications that require urgent solutions. Although conventional antibiotics limit bacterial biofilm formation, they ignore the bone loss caused by osteoclast formation during post-operative orthopaedic implant-related infections. Fortunately, enoxacin exerts both antibacterial and osteoclast inhibitory effects, playing a role in limiting infection and preventing bone loss. However, enoxacin lacks specificity in bone tissue and low bioavailability-related adverse effects, which hinders translational practice. Here, we developed a nanosystem (Eno@MSN-D) based on enoxacin (Eno)-loaded mesoporous silica nanoparticles (MSN), decorated with the eight repeating sequences of aspartate (D-Asp8), and coated with polyethylene glycol The release results suggested that Eno@MSN-D exhibits a high sensitivity to acidic environment. Moreover, this Eno@MSN-D delivery nanosystem exhibited both antibacterial and anti-osteoclast properties *in vitro*. The cytotoxicity assay revealed no cytotoxicity at the low concentration (20 μg/ml) and Eno@MSN-D inhibited RANKL-induced osteoclast differentiation. Importantly, Eno@MSN-D allowed the targeted release of enoxacin in infected bone tissue. Bone morphometric analysis and histopathology assays demonstrated that Eno@MSN-D has antibacterial and antiosteoclastic effects *in vivo*, thereby preventing implant-related infections and bone loss. Overall, our study highlights the significance of novel biomaterials that offer new alternatives to treat and prevent orthopaedic Staphylococcus aureus-related implantation infections and bone loss.

## 1 Introduction

Owing to the beneficial application of orthopaedic implants and improvement in treatments, patients affected by several orthopaedic diseases, including congenital malformations, acquired deformities, and osteoarthritis, as well as fractures can achieve satisfactory therapeutic effects. However, as a “double-edged sword”, the widespread use of implants is associated with new medical complications, and post-operative implant-related infections are an issue that needs to be addressed. The incidence of infection in orthopaedic surgery is approximately 5%, with post-operative infection rates of closed fractures making up 3.6–8.1%. In contrast, the incidence of open fracture is as high as 21.2% (Johnson et al., 2007). Antibiotics are still the primary treatment method for bone and joint infections; however, because of the limited penetration of antibiotics in bone tissues, sufficient blood concentrations cannot be achieved (Noukrati et al., 2016). Additionally, the lack of blood supply around the implant and the formation of bacterial biofilm (Lazzarini et al., 2004; Li et al., 2019), usually result in ineffective anti-infection treatments. This not only increases the duration of hospital stays and the total cost but also reduces the effectiveness of rehabilitation.

The pathogenesis of post-operative infections due to the presence of implants differs from that of general post-operative infections. The gap between the body tissue and the implant is a fibroinflammatory area where host immunity is suppressed, resistance is low, and bacteria can quickly colonise to form infections (Schierholz and Beuth, 2001). Besides, after bacterial invasion of the bone muscle system, due to intra-operative soft tissue injury and destruction of the blood supply, bacteria can colonise the proximities of the implants and form biofilms (Zhou et al., 2020). Bacterial biofilms limit antibiotic diffusion into the infected area, further reducing the concentration of antibiotics that can enter the infected area (Lynch and Abbanat, 2010; Buommino et al., 2014). Therefore, the efficient use of antibiotics is the key to limiting infections and inhibiting biofilm formation. Improving antibiotic treatment efficiency and increasing the concentration of drugs in bony tissues is particularly crucial for treating bone and joint infections. On the contrary, abnormal bone metabolism caused by bacterial erosion and bone destruction can activate a large number of osteoclasts, resulting in bone degradation and absorption (Dapunt et al., 2014; Oliveira et al., 2020). Simple anti-infection treatments of post-operative implant infections cannot eliminate the excessive osteoclast activation due to the inflammatory environment, which results in bone loss and bone destruction. The inhibition of osteoclast formation and bone resorption is an effective strategy to reduce bone loss and maintain long-term implant stability.

Enoxacin, a third-generation fluoroquinolone antibiotic, has a broad-spectrum and robust bactericidal effect. Additionally, enoxacin has an inhibitory effect on osteoclasts (Ostrov et al., 2009; Toro et al., 2012), and we previously reported that its mechanism is to occupy the ATP binding domain of the JNK protein, inhibit the phosphorylation of JNK, and activate the JNK/MAPKs signalling pathway (Liu et al., 2014). In terms of bacterial erosion of post-operative implant infections and the pathological state of a large number of osteoclasts activated in inflammatory environments, enoxacin exerts both antibacterial and anti-osteoclastic activities, making it an ideal drug candidate to prevent and treat post-operative implant-related infections. However, poor bioavailability due to its poor bone-targeting specificity and systemic toxicity (Nielsen and Low, 2020) limits enoxacin clinical applicability in treating orthopaedic implant-related post-operative infections.

In recent years, owing to their high specific surface area and large pore volume, mesoporous silica nanoparticles (MSNs) offer advantages that include excellent loading capability and biocompatibility, making them ideal candidates for drug delivery systems (Ravichandran et al., 2013; Zhou et al., 2015; Martínez-Carmona et al., 2018). Nevertheless, despite the promising applicability of MSNs for patients with post-operative infection related to orthopaedic endophytes, the lack of specific targeting to the infected bone tissue reduces the therapeutic effect of encapsulated antibiotics, simultaneously promoting drug resistance. Reportedly, eight repeating sequences of aspartate (D-Asp8) preferably bound to highly crystalline hydroxyapatite, and D-Asp8 could successfully bind to bone resorption surfaces to target osteoclasts (Liu et al., 2015). Based on these findings, to improve target specificity, we deployed MSNs as a carrier for the transport of enoxacin equipped with the bone-targeting D-Asp8 and coated with polyethylene glycol (PEG) to prevent premature release of enoxacin before reaching the target tissue. A bone-targeted delivery system containing enoxacin (Eno@MSN-D) was prepared for the targeted release of enoxacin in infected bone tissue. We hypothesised that Eno@MSN-D has antibacterial properties and can inhibit osteoclast activation, thereby preventing *Staphylococcus aureus*-related implantation infections and consequent bone loss.

## 2 Materials and Methods

### 2.1 Materials

Hexadecyl trimethyl ammonium bromide (CTAB), tetraethyl orthosilicate (TEOS), silane-polyethylene glycol-carboxyl (Silane-PEG-COOH), MES buffer, aqueous ammonia, hydrochloric acid, ethanol, ethyl acetate, crystal violet solution, phosphate-buffered saline (PBS), 1-(3-dimethylaminopropyl)-3-ethyl carbodiimide hydrochloride (EDC), and N-hydroxy succinimide (NHS) were obtained from Sinopharm Chemical Reagent Co., China. Enoxacin was obtained from Sigma Aldrich, St. Louis, United States. Dialysis bags were obtained from UC Union Carbide, Danbury, United States. *Staphylococcus aureus* (ATCC25923), *S. epidermidis* (ATCC12228), and methicillin-resistant *S. aureus* (ATCC43300) were obtained from American Type Culture Collection, Manassas, VA, United States. Trypsin soy broth (TSB) and trypsin soybean agar plate was obtained from Shanghai Chengsheng Biotechnology Co., Ltd.

### 2.2 MSN Synthesis

MSN were synthesised as previously reported, with minor modifications (Lee et al., 2010). NaOH aqueous solution (2 M, 0.35 ml) was mixed with water (50 ml) containing CTAB (100 mg), and the solution was heated to 70°C while stirring. Thereafter, TEOS (0.5 ml) was introduced dropwise to the reaction mixture. After 3 min, ethyl acetate (0.5 ml) was added, and the mixture was stirred for 30 s, followed by an ageing procedure at 70°C for 2 h. The precipitate was collected by centrifugation and washed with ethanol. The collected products were extracted for 6 h using a solution of hydrochloric acid (HCl) in ethanol (10% v/v) at 78°C by refluxing to remove CTAB and obtain the MSNs.

For enoxacin loading, 50 mg of MSN was dispersed in 10 ml of methanol solution mixed with enoxacin (25 mg), and the mixture was shaken at 25°C for 4 h. The dispersion solution was then centrifuged at 10,000 rpm to collect the enoxacin-loaded MSN (Eno@MSN). Next, the Eno@MSN were washed with distilled water to remove enoxacin from the exterior surface.

### 2.3 Synthesis of Eno@MSN-D

**FIGURE 1:**
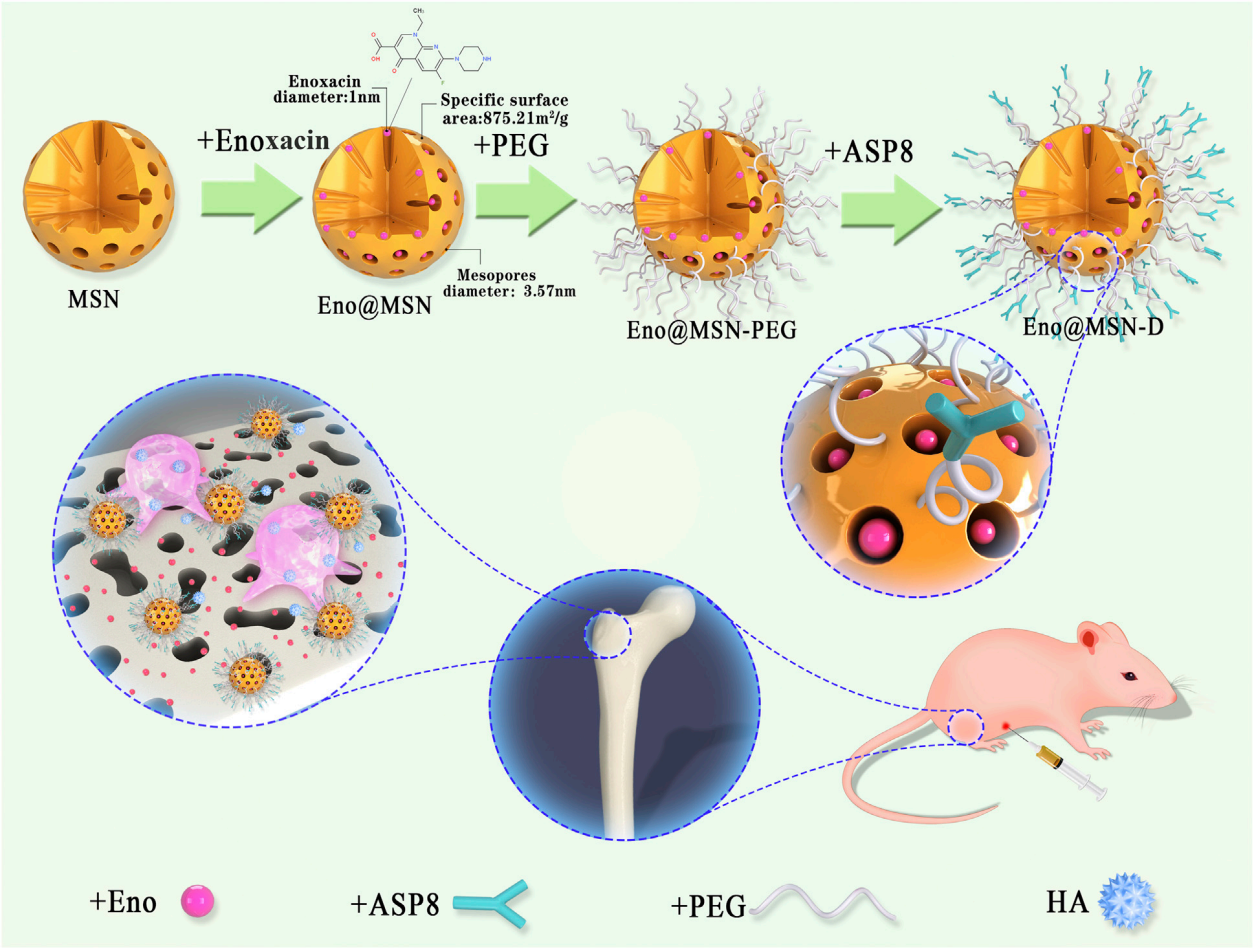
Schematic diagram of the process flow for synthesising Eno@MSN-D nano slow-release particles based on mesoporous silica nanoparticles (MSN) and schematic diagram of the action mechanism of Eno@MSN-D nanoparticles.

Briefly, Eno@MSN was dispersed in 10 ml of water mixed with Silane-PEG-COOH (28 mg). Thereafter, 0.2 ml of aqueous ammonia was added to the mixture and stirred for the next 4 h. The dispersion solution was centrifuged at 10,000 rpm, and the nanoparticles were washed with water three times to obtain Eno@MSN-PEG. Next, 30 mg of Eno@MSN-PEG was dispersed in 5 ml of MES buffer, and then 5.0 mg of EDC and 3.8 mg of NHS were added to the solution. After the excitation reaction for 30 min, 5 mg of D-Asp8 moiety was added to the mixture and reacted for 2 h. Subsequently, the solution was then centrifuged at 10,000 rpm and washed with water three times to obtain Eno@MSN-D. Finally, to monitor the cellular uptake of Eno@MSN-D by confocal microscopy, the red fluorescent dye Cy7 was loaded into Eno@MSN-D for tracking and labelling. Figure 1 shows a scheme of Eno@MSN-D production and its mechanism.

### 2.4 Characterisation of Nanoparticles

Transmission electron microscopy (TEM) images were obtained using a JEM2010 instrument (JEOL, Tokyo, Japan) with an acceleration voltage of 200 kV to investigate the ultrastructure of drug-free MSN, Eno@MSN, and Eno@MSN-D. Nitrogen adsorption–desorption was measured using a Micromeritics porosity analyser (Micromeritics, Norcross, GA). For the surface area, pore volume was calculated using the Brunauer–Emmett–Teller (BET) and Barrett–Joyner–Halenda (BJH) analyses, respectively. Additionally, the hydrodynamic size of the samples was measured by dynamic light scattering (DLS) using a Zetasizer Nano instrument (Malvern Instruments, Malvern, United Kingdom) at 298 K. Finally, the thermal stability and composition of the solid samples were determined by TGA. TGA was performed on a TG 209F1 thermal analyser (NETZSCH, Germany) at a heating rate of 10°C/min in a continuously moving N_2_ atmosphere.

### 2.5 Drug Release Determination

The *in vitro* pH-sensitive enoxacin release pattern of Eno@MSN-D was evaluated in PBS of pH 7.4 and 6.0. The Eno@MSN-D dispersion solution was loaded in a dialysis bag with a molecular weight cut-off of 8 kDa. The dialysis bag was placed in 8 ml of PBS and gently shaken at 37°C. One millilitre of the supernatant was collected at predetermined time points, and the amount of drug released was determined using an ultraviolet spectrophotometer at *λ* = 232 nm. Thereafter, 1 ml of fresh PBS was added to the dialysate to maintain a constant volume.

### 2.6 Bacterial Strains and Cultures

*Staphylococcus aureus* (ATCC25923), *S. epidermidis* (ATCC12228), and methicillin-resistant *S. aureus* (ATCC43300) were stored on a TSA plate at −4°C. The three bacterial colonies in a centrifuge tube containing TSB were cultured overnight at 37 °C in an oscillator incubator at 150 rpm. Subsequently, the samples was centrifuged at 5000 rpm for 5 min. After centrifugation, the supernatant was discarded. TSB was used to adjust the bacterial precipitation concentration to 1 × 10^6^ CFU/ml for storage.

### 2.7 Minimum Inhibitory Concentration

To each well of 96-well plates, 100 μL of ATCC25923, ATCC12228, and ATCC4330 bacteria cells (1 × 10^6^ CFU/ml). Different concentration gradients of TSB (as the blank control), Eno, and Eno@MSN-D were separately added into the 96-well plates. After incubating at 37°C for 24 h, the bottom of the plate was closely inspected for biofilm formation. The lowest concentration without biofilm formation was determined as MIC, and it was determined using a microtiter plate dilution assay.

### 2.8 Determination of Bacterial Biofilm Formation

A crystal violet assay was performed using 96-well microtiter plates to observe the effects of Eno@MSN-D on bacterial biofilm formation. Briefly, ATCC25923 with a concentration of 1 × 10^6^ CFU/ml were added to 96-well plates at 100 µl/well in triplicate and cultured for 24 h in 37°C. Then, each well of plates was added 100 μl of TSB (10 μg/ml), MSN (10 μg/ml), Eno (10 μg/ml), L-Eno@MSN (5 μg/ml), and H-Eno@MSN-D (10 μg/ml). Next, each well was washed with PBS three times and stained with 0.1% crystal violet solution. The plates were then incubated at room temperature for 15 min and then gently washed with PBS three times to remove excess crystal violet. Afterward, 33% of acetic acid was added to each well and placed in the incubator at 37°C for 30 min. The absorbance at 570 nm was determined using a microplate reader.

### 2.9 Morphological Characterisation of Bacteria and Bacterial Biofilm

The morphology of bacteria and the formation of biofilm were observed by SEM. ATCC25923 with a concentration of 1 × 10^6^ CFU/ml was added to 12-well plates at 1ml/well in triplicate and cultured for 24 h in 37°C. TSB (10 μg/ml), MSN (10 μg/ml), and Eno@MSN-D (5, 10 μg/ml) were added to 12-well plates at the same time, and cover slides were placed in the wells. Next, the cover slides were gently washed with PBS and fixed in 2.5% glutaraldehyde for 12 h. After washing with PBS, the sample was dehydrated in an ethanol series (50, 60, 70, 80, 90, and 100%). After freeze-drying and gold-sputtering the samples, they were observed by SEM (SU8010, Hitachi, Japan).

### 2.10 Determination of Antibacterial Properties of Eno@MSN-D *in vitro*

The effects of Eno@MSN-D on bacterial biofilm were observed using a fluorescence inversion microscope system. ATCC25923 was diluted to 1 × 10^6^ CFU/ml by TSB, and 1 ml was placed in a confocal Petri dish with a glass bottom and incubated at 37°C for 24 h. After incubation, the supernatant in the dish was removed, and the biofilm was treated with five or 10 μg/ml Eno@MSN-D. The control group included MSN (10 μg/ml) and TSB (10 μg/ml). The Petri dishes were then washed with aseptic PBS to remove loosely bound bacteria. Bacteria in the biofilm were stained with a LIVE/DEAD^®^ BacLight bacterial viability kit (L7007) at room temperature for 15 min in the dark. The dye was cleaned with aseptic PBS, and the biofilm was observed using a fluorescence inversion microscope. Bacteria with intact and damaged cell membranes were obtained by scanning under excitation using the green (488 nm) and red (543 nm) channels, respectively.

### 2.11 Cell Viability and Osteoclast Differentiation Assay *in vitro*

To evaluate the cytotoxic effect of Eno@MSN-D, we used Cell Counting Kit-8 (CCK-8, Dojindo Molecular Technologies, Inc, Kumamoto, Japan) according to the manufacturer’s instructions. Briefly, bone marrow macrophages (BMMs) were added to 96-well plates at 8 × 10^3^ cells/well in triplicate and cultured for 24 h in alpha modification of Eagle’s medium (*α*-MEM, Gaithersburg, MD, United States) containing 30 ng/ml macrophage colony-stimulating factor (M-CSF; PeproTech, Rocky Hill, NJ, United States), 10% foetal bovine serum (FBS; Gibco-BRL, Sydney, Australia), and 1% penicillin/streptomycin. BMMs were then separately treated with different concentrations of Eno@MSN-D (0, 0.625, 1.25, 2.5, 5, 10, 20, 40, 80 and 160 μg/ml) for 48 or 96 h. Next, 10 µL of CCK-8 substrate was added to each well, and the plate was incubated at 37°C under 5% CO_2_ for 2 h. The absorbance of sample in each well was measured at 450 nm using an ELX800 microplate reader (Bio-Tek Instruments Inc, Winooski, VT, United States).

BMMs were seeded in a 96-well plate at a density of 8 × 10^3^ cells/well in *α*-MEM with 30 ng/ml M-CSF, 50 ng/ml RANKL (PeproTech, Rocky Hill, NJ, United States), and different concentrations of Eno@MSN-D (0, 2.5, 5, and 10 μg/ml). BMMs were supplemented with fresh medium every 2 days until mature osteoclasts were observed. Next, the cells were fixed with 4% paraformaldehyde and stained for tartrate-resistant acid phosphatase (TRAP; Sigma Aldrich, St. Louis, MO, United States) activity. The mature osteoclasts (TRAP-positive cells with ≥3 nuclei) were counted, and their spread area was measured.

### 2.12 Bone-Targeting Properties of Eno@MSN-D *in vivo*

All animal experiments were performed in the Department of Animal Experimental Sciences of Nanchang University, under the approval and guidance of the Animal Experimental Ethics Committee of the First Affiliated Hospital of Nanchang University. Targeting of common fluorescent MSN and fluorescent Eno@MSN-D was evaluated in Sprague–Dawley (SD) rats [Shanghai SLAC Laboratory Animal Co., Ltd (China)]. Eighteen 3-month-old female SD rats were divided into 3 groups: MSN control group, PEG-MSN group, and Eno@MSN-D experimental group. Six animals from each group were injected in the tail vein with identical doses of nanoparticles (50 mg/kg body weight). After injection, all animals had free access to food and water. The animals were euthanised after 4 and 72 h, and the main organs (heart, liver, spleen, lung, kidney, femur) were removed. A fluorescence imaging system was used to detect the fluorescence of each organ in each group.

### 2.13 Antibacterial Properties of Eno@MSN-D *in vivo*

Fifty specific pathogen-free grade 12-week-old female SD rats were used and randomly assigned to five independent groups. All animals were housed in clean plastic cages with a 12-h light/dark cycle and free access to fresh food and water. The rats in the five groups were anaesthetised by intraperitoneal injection with 10% chloral hydrate (4 ml/kg). After complete anaesthesia, the rats were placed in the supine position to remove hair from the left knee joint, subsequently sterilised with 75% ethanol. A 15-mm incision was made along the lateral end of the femur. Subcutaneous tissues and muscles of the lateral femoral condyle were incised, the joint capsule and lateral collateral ligaments were retained, and the femoral condyle was fully exposed. The bone marrow cavity of the femur was opened and expanded to a depth of 10 mm using an electric drill of diameter 1 mm. Subsequently, a 1-mm diameter titanium rod of length 10 mm was implanted. The ATCC25923 concentration was set to 1 × 10^6^ CFU/ml, and 100 µl was injected into the bone marrow cavity. The hole in the femoral condyle was blocked with bone wax. A saline solution was used for flushing the wound, and a medical suture was used to close the wound. Berberine was then applied to the wound. In the Sham group, only the condyle of the femur was exposed before closing the incision. After the operation, the rats were resuscitated under a fan heater and put back in their cages. The rats were maintained in separate cages; they had free access to food and water.

In the first week post-operation, body temperature and weight were examined every day. After 1 week of observation, each experimental group was injected with different drugs. Group A: Sham group (normal saline); group B: NS group (normal saline); group C: MSN group (50 mg/kg body weight); group D: Eno group (4 mg/kg body weight); group E: Eno@MSN-D group (50 mg/kg body weight). Body weight and temperature were recorded every 3 days. The drugs were then intraperitoneally injected every day for a total of 4 weeks, and the animals were euthanised 4 weeks later. The femurs were separated from the skin and subcutaneous tissue under aseptic conditions. Soft tissues were removed, and the femurs were prepared for further experiments. All titanium rods were collected and processed for analysis.

Bacteria attached to the titanium rods were detected by SEM, fluorescence staining, plate colony counting method, and plasma coagulase test. After removing the titanium rods from the distal femur and washing with PBS, 5 bars of each group were randomly placed in 2.5% glutaraldehyde for 12 h. Subsequently, they were dehydrated with an ethanol series (50, 60, 70, 80, 90, and 100%). The samples were freeze-dried and gold-sputtered. The surfaces of the titanium rods were then observed by SEM. Five titanium rods were randomly selected and fixed for 12 h as described above. After washing with PBS, the rods were stained using the LIVE/DEAD ®BacLight bacterial viability kit (L7007) at room temperature for 15 min in the dark. They were then observed using a fluorescence inversion microscope. Meanwhile, the titanium rods were washed, placed in 1 ml of TSB, and ultrasonicated for 15 min. After a 10-fold dilution, 100 µl of suspension was evenly applied to a TSA plate and incubated at 37°C for 24 h. The CFU was calculated according to the colony count on the plates. Additionally, a plasma coagulase test was performed to determine whether the bacteria attached to the titanium rods were *S. aureus*. A single colony was picked from the TSA plate and suspended in 50 µl of PBS before adding 50 µl of rabbit plasma. The occurrence of agglutination indicated that the bacteria were *S. aureus*.

### 2.14 Micro-Computed Tomography

After euthanasia, the femur from the rats of each group was removed completely under sterile conditions. After removing the titanium rod, the femur was fixed with 4% paraformaldehyde for 2 days and washed with tap water for 24 h. The peripheral bone structure of the distal femoral implant was then evaluated 28 days after the injection of different drugs. The femurs were examined using a desktop micro-X-ray computed tomography system (micro-CT Skyscan1076, Aartselaar, Belgium) equipped with a 40 kV X-ray source and 12.60 µm camera pixel size. A reconstructed dataset with an image pixel size of 18.26 µm was generated *via* scanning. To determine the axial trabecular volume of interest, we selected a region of interest (ROI) of length 5.578 mm closest to the growth plate edge. Micro-CT images of the transverse, sagittal, and coronal sections of the area around the implants were obtained. Object volume, total VOI volume, bone evolution fraction, bone mineral density, trabecular thickness, trabecular spacing, and trabecular number were used as indices to measure trabecular bone mass and its distribution.

### 2.15 Histology and Histomorphometry

Bone histology was used to assess infection and bone structure changes around the distal femoral implants. The femurs of the rats were fixed with 4% paraformaldehyde at room temperature for 24 h. Subsequently, 10% of ethylenediamine tetra-acetic acid was fully decalcified and dehydrated with an ethanol series (50, 75, 80, 85, 90, 95, and 100%). The bone tissue, after transparent treatment in xylene, was embedded in paraffin. The sample was longitudinally cut into 5-μm-thick slices. After slicing, histological sections were prepared for TRAP and HE staining. The slices were observed with an optical microscope. Representative images were randomly obtained from the distal femur implanted with titanium rods. We used Image-Pro Plus 6.0 software (Media Cybernetics, MD, United States) to process the images of TRAP-stained sections and counted the number and determined the area of osteoclasts per field of view.

### 2.16 Statistical Analysis

IBM SPSS statistics 22 (SPSS Inc, United States) software was used for statistical analysis. The results are presented as mean ± SD. Experiments were conducted at least three times. One-way analysis of variance (ANOVA) with Bonferroni post hoc test was performed to determine group differences. Results with *p* < 0.05 were considered statistically significant.

## 3 Results

### 3.1 Synthesis and Characterisation of Eno@MSN-D

The protocol for the preparation of Eno@MSN-D is shown in Figure 1. Nanoparticle mesoporous carriers were prepared using TEOS as a hydrolytic inorganic precursor and the surfactant CTAB as the poreogenic substance. Bare MSN was obtained by solvent extraction of surfactant removal and was then loaded with Eno by free diffusion. Subsequently, PEG was grafted onto silica surfaces and channels to act as a gatekeeper for drug delivery. Finally, Eno@MSN-PEG was constructed by fusing D-Asp8 to Eno@MSN-D.

**FIGURE 2:**
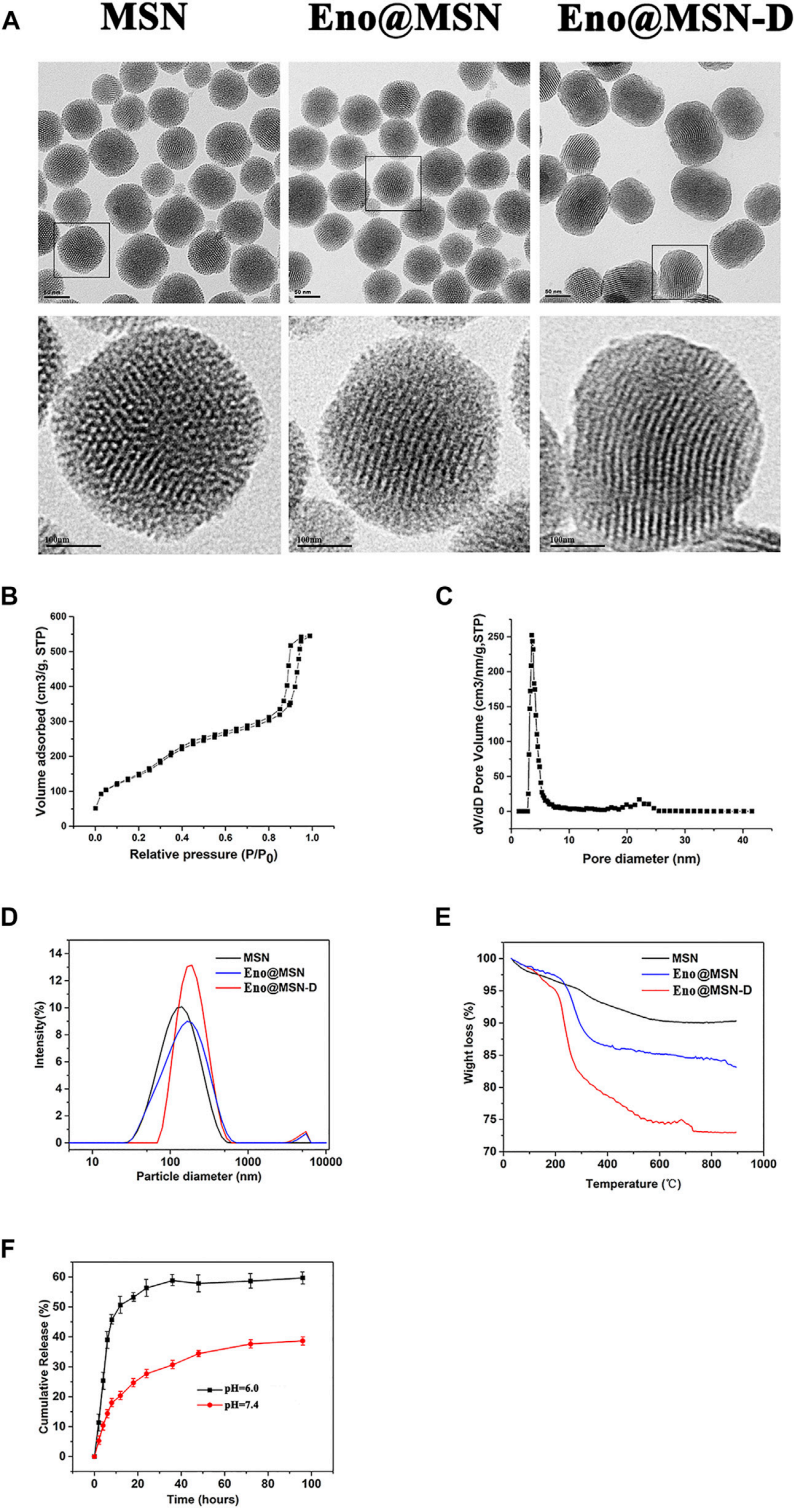
**(A)** Typical TEM images of MSN, Eno@MSN, and Eno@MSN-D.**(B)**. N_2_ adsorption−desorption isotherms curves of the MSN. **(C)** Pore size distribution curves of the MSN. **(D)** The dynamic light scattering (DLS) curves of MSN, Eno@MSN, and Eno@MSN-D. **(E)** TGA curves recorded for MSN, Eno@MSN, and Eno@MSN-D. **(F)** Accumulative release curve of Eno@MSN-D in different pH PBS (*p*H = 7.4, 6.0).

From the TEM images (Figure 2A), we observed the size and morphological characteristics of the three nanoparticle types. MSN maintained a highly ordered mesoporous structure, which disappeared after enoxacin loading and PEG/D-Asp8 immobilisation, and the average particle size of Eno@MSN-D was also larger than that of MSN and Eno@MSN, consistent with the results obtained in the DLS analysis (Figure 2D). The average particle diameters of the MSN, Eno@MSN, and Eno@MSN-D were 113.9, 133.8, and 179.7 nm, respectively. Particle dispersion index (PDI) and zeta potential of the nanoparticles were obtained using the DLS analysis. The PDI of MSN, Eno@MSN, and Eno@MSN-D was 0.213, 0.272, and 0.202 nm and their zeta potential was −24.3, −22.4, and −19.3 mV, respectively.

The nitrogen adsorption-desorption isotherm curves further exhibited the classical Langmuir type IV isotherm, indicating the presence of a mesoporous structure in the MSN (Figure 2B). Furthermore, the capillar condensation in the range of 0.8–1.0 refers to large pores and the adsorption branch was used for the analysis of the pore size distribution (Figure 2C). The specific surface area and pore size of MSN were 875.21 m^2^/g and 3.57 nm, respectively. In addition, Figure 2E shows the TGA curves of all nanoparticles, indicating that during analysis, the weight loss of MSN, Gen@MSN, and Eno@MSN-D was close to 7.71, 15.62, and 25.65%, respectively.

### 3.2 Drug Loading and Release *in vitro*

It is well known that controlled release performance is an indispensable key step for the expected nanoparticles. Under simulated physiology conditions (*p*H = 7.4) and acid microenvironment caused by implant infection (*p*H = 6.0), two different pH buffer solutions were simulated for pH-response release pattern. In Eno@MSN-D, burst release was not found after stirring for 150 min at pH 7.4, and approximately 40% of enoxacin was released (Figure 2F). Interestingly, when the pH was decreased from 7.4 to 6.0, the cumulative release of enoxacin increased to approximately 60% (Figure 2F).

### 3.3 Antibacterial Properties of Eno@MSN-D *in vitro*

As previously reported, the MIC values of enoxacin against *S. aureus* (ATCC25923), *S. epidermidis* (ATCC12228), and methicillin-resistant *S. aureus* (ATCC43300) were 2, 2, and 4 μg/ml, respectively (Nie et al., 2017). Here, the MIC values of Eno@MSN-D against ATCC25923, ATCC12228, and ATCC43300 were 4, 4, and 8 μg/ml, respectively.

**FIGURE 3:**
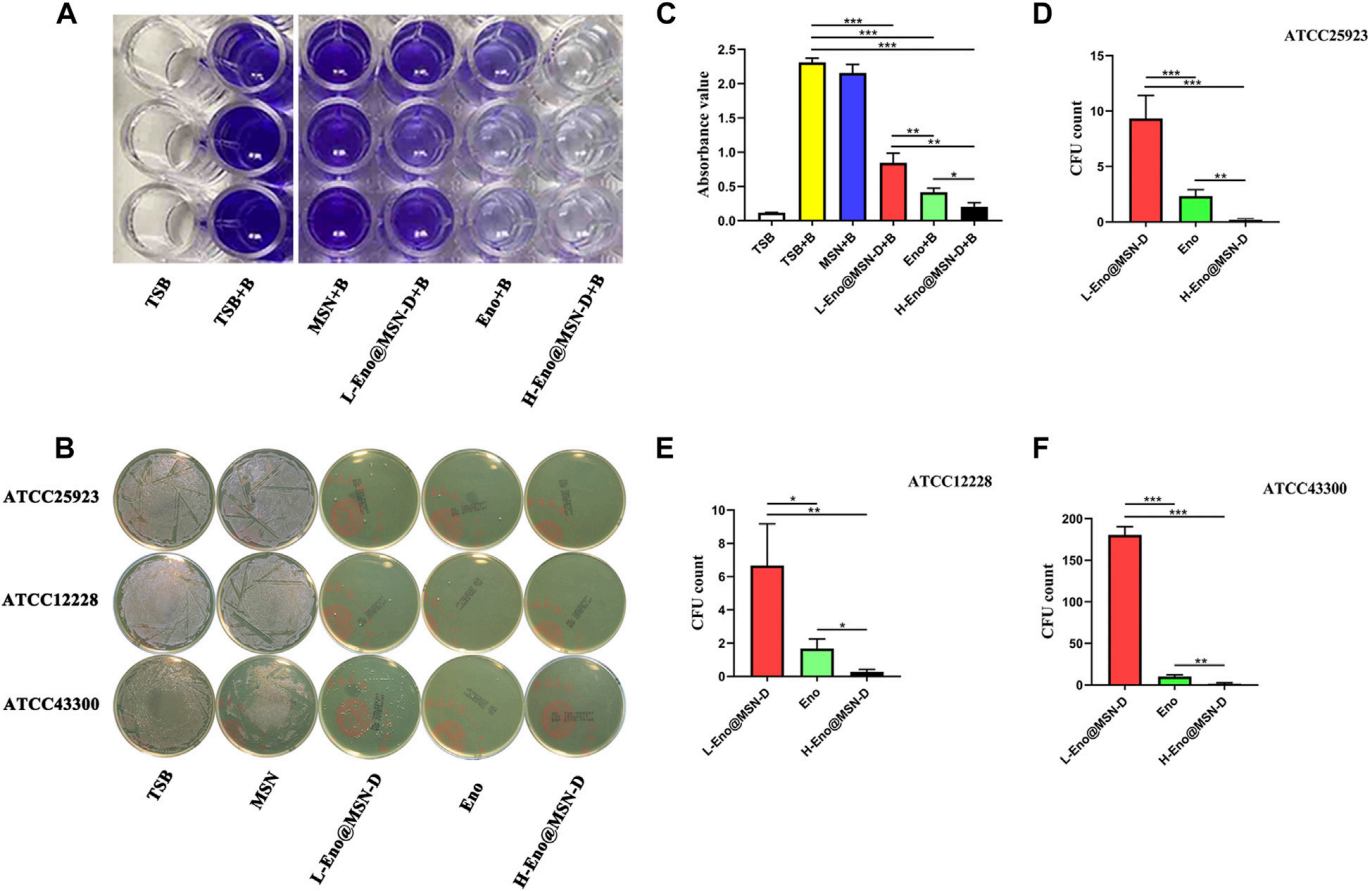
**(A–C)** TSB (10 μg/ml), MSN (10 μg/ml), Eno (10 μg/ml), L-Eno@MSN (5 μg/ml), and H-Eno@MSN-D (10 μg/ml) were co-cultured with bacteria to form a biofilm, and then the absorbance was measured by crystal violet staining. **(B)** Determination of antimicrobial properties bacterial using the colony counting plate method. **(D)** The number of CFU after co-culture of ATCC25923 with Eno@MSN-D. (E) The number of CFU after co-culture of ATCC12228 with Eno@MSN-D. **(F)** The number of CFU after co-culture of ATCC43300 with Eno@MSN-D.

ATCC25923 was co-cultured with different concentrations of Eno@MSN-D for 24 h, and the absorbance of the samples was measured by crystal violet staining to determine the amount of biofilm formation (Figure 3A). We established a TSB blank control group, TSB and bacteria (TSB + B) co-culture-positive control group, 10 μg/ml MSN and bacteria (MSN + B) co-culture-positive control group, 10 μg/ml enoxacin and bacteria (Eno + B) co-culture-positive group, low concentration of 5 μg/ml Eno@MSN-D and bacteria (L-Eno@MSN-D + B) co-culture-positive group, and high concentration of 10 μg/ml Eno@MSN-D and bacteria (H-Eno@MSN-D + B) co-culture-positive group. Our results showed that biofilm formation was significantly lower in the TSB groups than in the TSB + B groups. However, both Eno@MSN-D + B and Eno + B groups showed decreased biofilm formation after treatment. Compared to the group treated with L-Eno@MSN-D + B, superior antibacterial effects were observed in the H-Eno@MSN-D + B group. Additionally, the H-Eno@MSN-D + B groups showed superior effects compared with the Eno + B groups, and there was a significant difference between them (Figure 3C). This result indicates that the bacterial biofilm formation decreased with Eno@MSN-D concentration, which further proved that the synthesised Eno@MSN-D has an antibacterial effect.

The antibacterial ability of Eno@MSN-D against each strain was verified using the bacterial colony counting plate method. ATCC25923, ATCC12228, and ATCC43300 were co-cultured with TSB, MSN, enoxacin, L-Eno@MSN-D, and H-Eno@MSN-D. Figures 3B,D–F shows no colony formation at the H-Eno@MSN-D concentration of 10 μg/ml. At the L-Eno@MSN-D concentration of 5 μg/ml, only a few colonies were formed in ATCC25923 and ATCC12228, while there were relatively more colonies in ATCC43300. However, many colonies were formed in the MSN and TSB groups. Interestingly, compared with the Eno group, the L-Eno@MSN-D group had a higher CFU number, whereas the H-Eno@MSN-D group showed fewer CFUs. In summary, Eno@MSN-D has apparent antibacterial effects.

**FIGURE 4:**
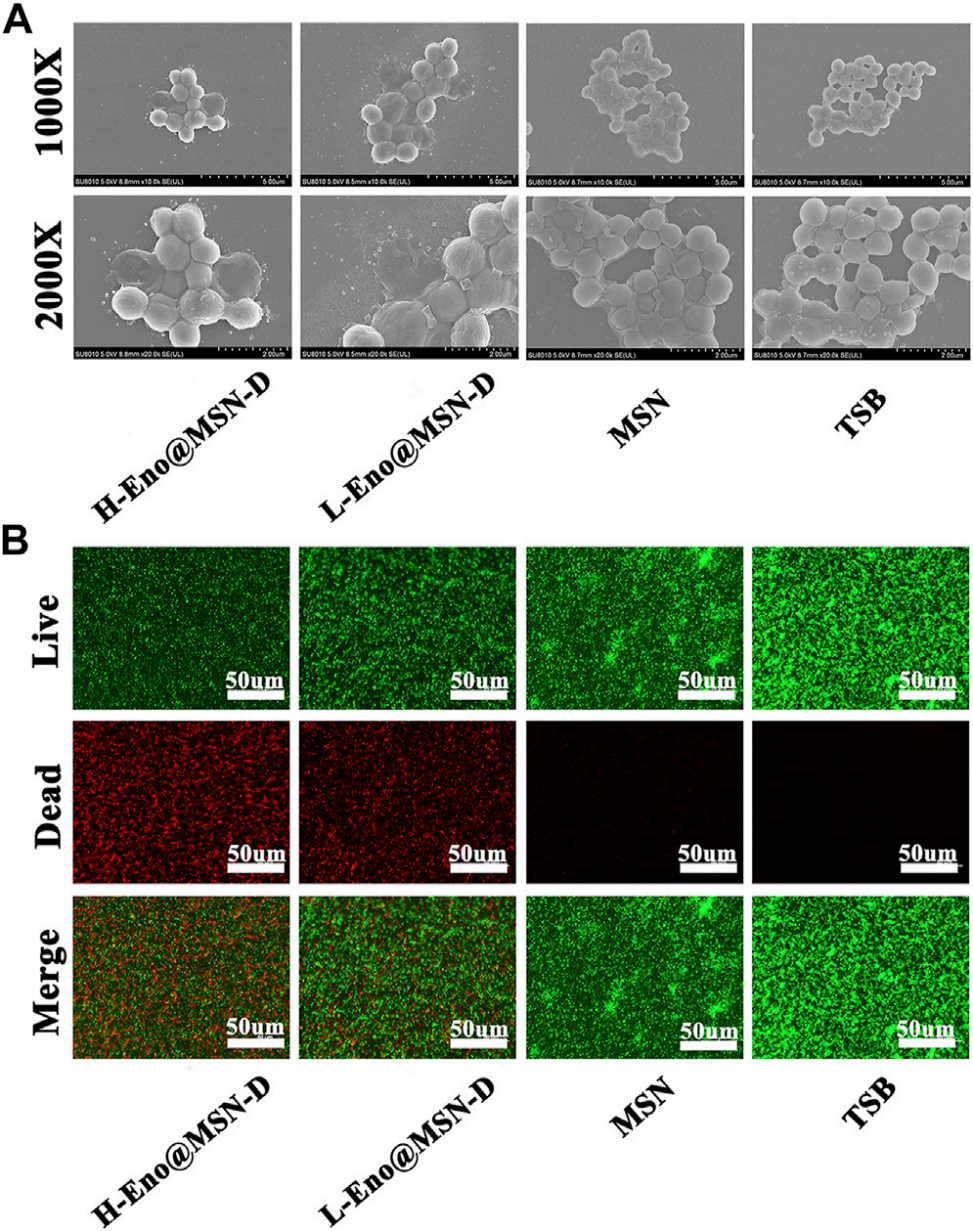
**(A)** SEM images of TSB (10 μg/ml), MSN (10 μg/ml), Eno (10 μg/ml), L-Eno@MSN (5 μg/ml), H-Eno@MSN-D (10 μg/ml), and bacteria co-cultured on cover slides. **(B)** The fluorescence inverted microscope images of TSB (10 μg/ml), MSN (10 μg/ml), Eno (10 μg/ml), L-Eno@MSN (5 μg/ml), H-Eno@MSN-D (10 μg/ml), and bacteria co-cultured on confocal Petri dishes.

We observed the surface of treated glass slides by SEM. Figure 4A shows that the integrity of some bacterial morphology was disrupted in the L-Eno@MSN-D and H-Eno@MSN-D groups. In the MSN and TSB groups, there was a significant adhesion between the bacteria, indicating the formation of biofilm. This observation also shows that Eno@MSN-D could destroy bacterial integrity, playing an antibacterial role, and inhibiting biofilm formation.

Live and dead bacteria were marked by green and red fluorescence, respectively. From the fluorescence inverted microscope pictures (Figure 4B), it is evident that with increasing drug concentrations of Eno@MSN-D, the red fluorescence of dead bacteria gradually increased. In contrast, the corresponding green fluorescence gradually weakened.

### 3.4 Eno@MSN-D Suppressed RANKL-Induced OC Differentiation Without Any Cytotoxicity *in vitro*

**FIGURE 5:**
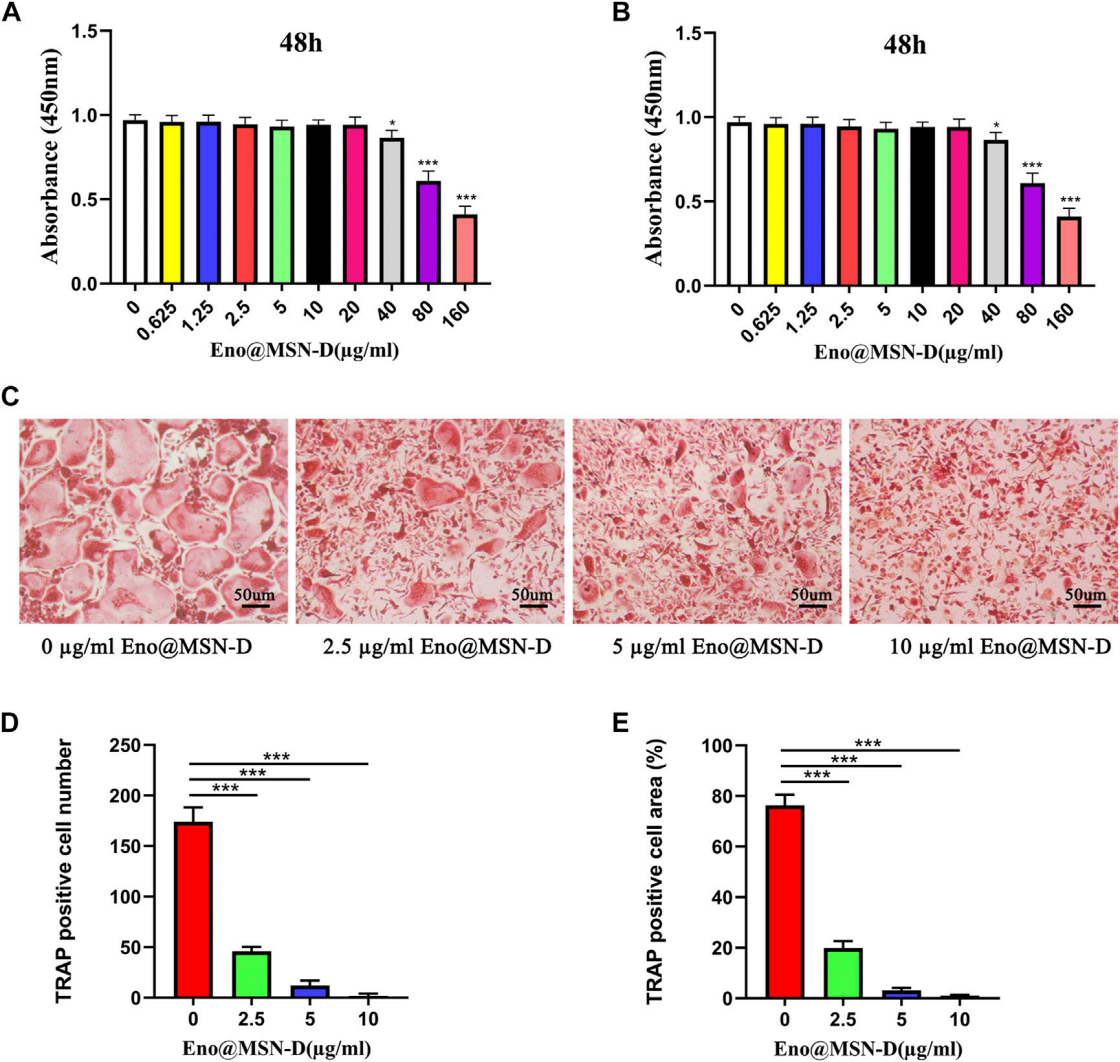
**(A,B)** BMMs were plated in 96-well plates and stimulated with M-CSF (30 ng/ml) and different concentrations of Eno@MSN-D (0–160 μg/ml) for 48 or 96 h. Cell viability was measured using the CCK-8 assay. **(C)** Bone marrow macrophages (BMMs) were treated with various concentrations of Eno@MSN-D (0, 2.5, 5, and 10 μg/ml) followed by M-CSF and RANKL stimulation for 5 days. The cells were then fixed with 4% paraformaldehyde and subjected to tartrate-resistant acid phosphatase (TRAP) staining. **(D,E)** The number and spread area of TRAP-positive multinuclear cells were determined.

Previously, we reported that enoxacin could inhibit osteoclast differentiation and function (Liu et al., 2014). However, the effect of Eno@MSN-D on the formation of osteoclasts needs to be explored. BMMs were stimulated with M-CSF and RANKL in the presence of different concentrations of Eno@MSN-D (0, 2.5, 5, and 10 μg/ml). Interestingly, the BMMs treated with Eno@MSN-D showed a significant concentration-dependent decrease in mature osteoclast formation (Figures 5C–E). Additionally, to determine whether the inhibitory effects of Eno@MSN-D were due to cytotoxicity, we used the CCK-8 assay to measure the effect of Eno@MSN-D on BMM proliferation and survival. Our data show that cell viability will not be significantly affected when the concentration is lower than 20 μg/ml Eno@MSN-D (Figures 5A,B).

### 3.5 Bone-Targeting Properties of Eno@MSN-D *in vivo*

**FIGURE 6:**
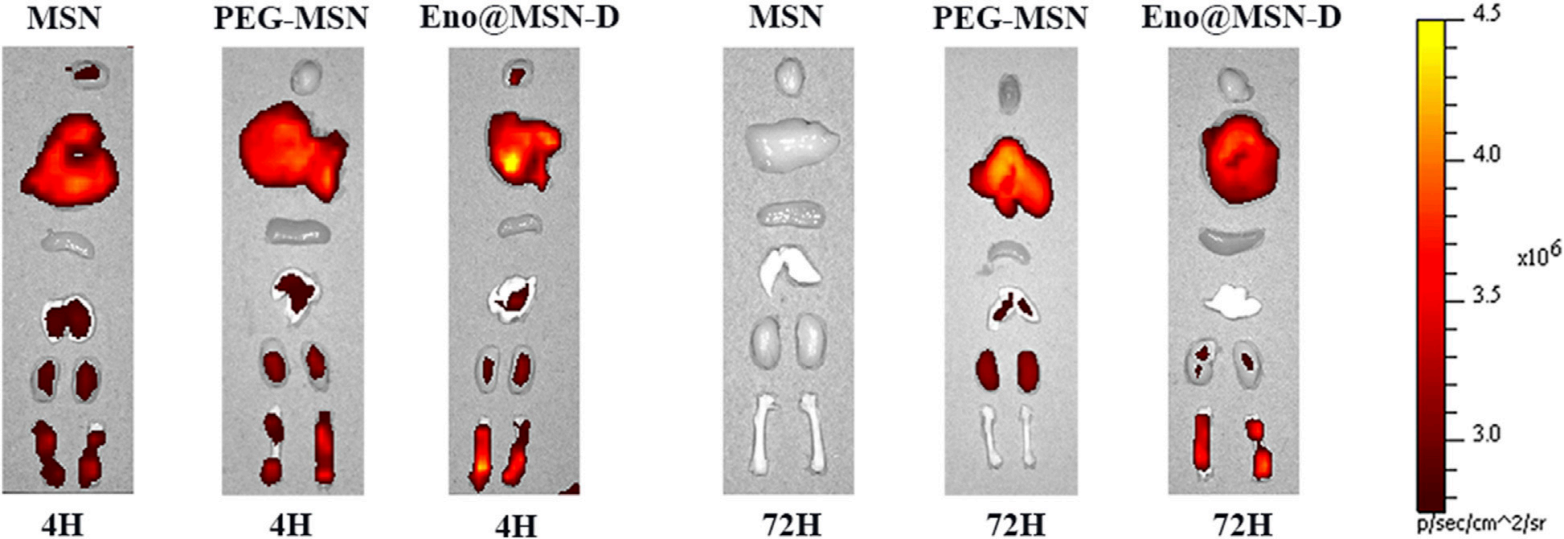
*In vivo* targeted fluorescence experiment: fluorescence diagrams of various organs 4 and 72 h after the injection of MSN, PEG-MSN, and Eno@MSN-D nanoparticles.

As shown in Figure 6, after 4 h, the fluorescence signals of the different organs in the MSN, PEG-MSN, and Eno@MSN-D group were almost the same. The MSN group was metabolised cleanly by the liver and kidney. However, the PEG-MSN and Eno@MSN-D group had a strong liver and kidney fluorescence signal after 72 h. More importantly, only the Eno@MSN-D group showed stronger fluorescence signals in the femur after 72 h, indicating that Eno@MSN-D also accumulated in the femur and exhibited bone-targeting abilities *in vivo*.

### 3.6 Antibacterial Properties of the Eno@MSN-D *in vivo*

**FIGURE 7:**
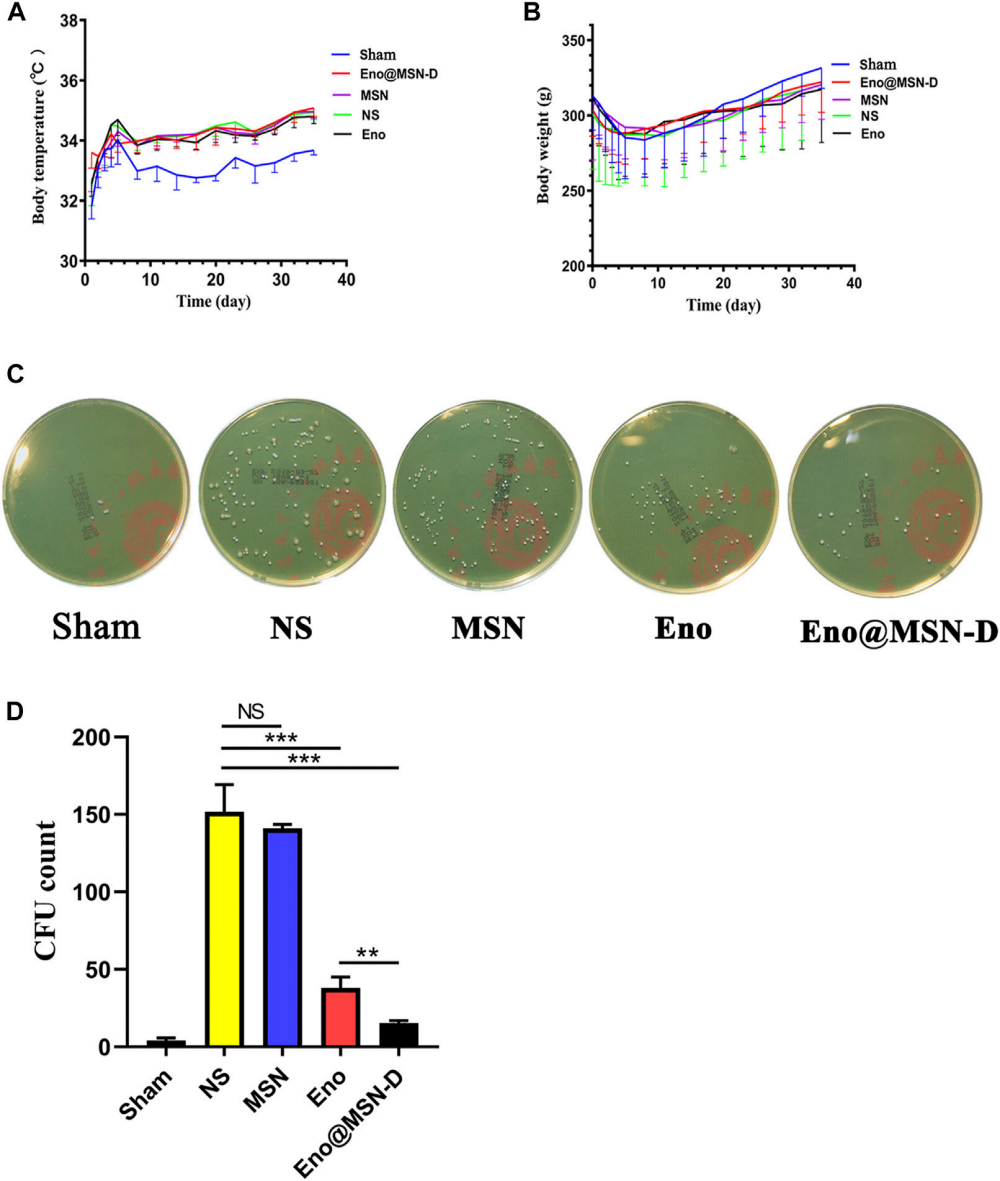
**(A)** Curve of body temperature change in rats. **(B)** Curve of body weight change in rats. **(C)** Plate colony count after ultrasonic concussion of bacteria on the surface of a titanium rod. **(D)** Statistical chart of plate colony count after the ultrasound treatment.

We determined the animal model infection by measuring the rats’ body temperature and weight over 5 weeks post-operation. As shown in Figures 7A,B, the bodyweight of rats in each group gradually decreased within 1 week after the operation, whereas the body temperature gradually increased. One week later, rat bodyweight began to increase in each group, whereas the Sham group body temperature began to decrease. However, the body temperature of the experimental groups did not change. There was no significant difference in body weight between the experimental and Sham groups (*p* > 0.05), but the body temperature differed between the experimental and Sham groups (*p* < 0.0001).

**FIGURE 8:**
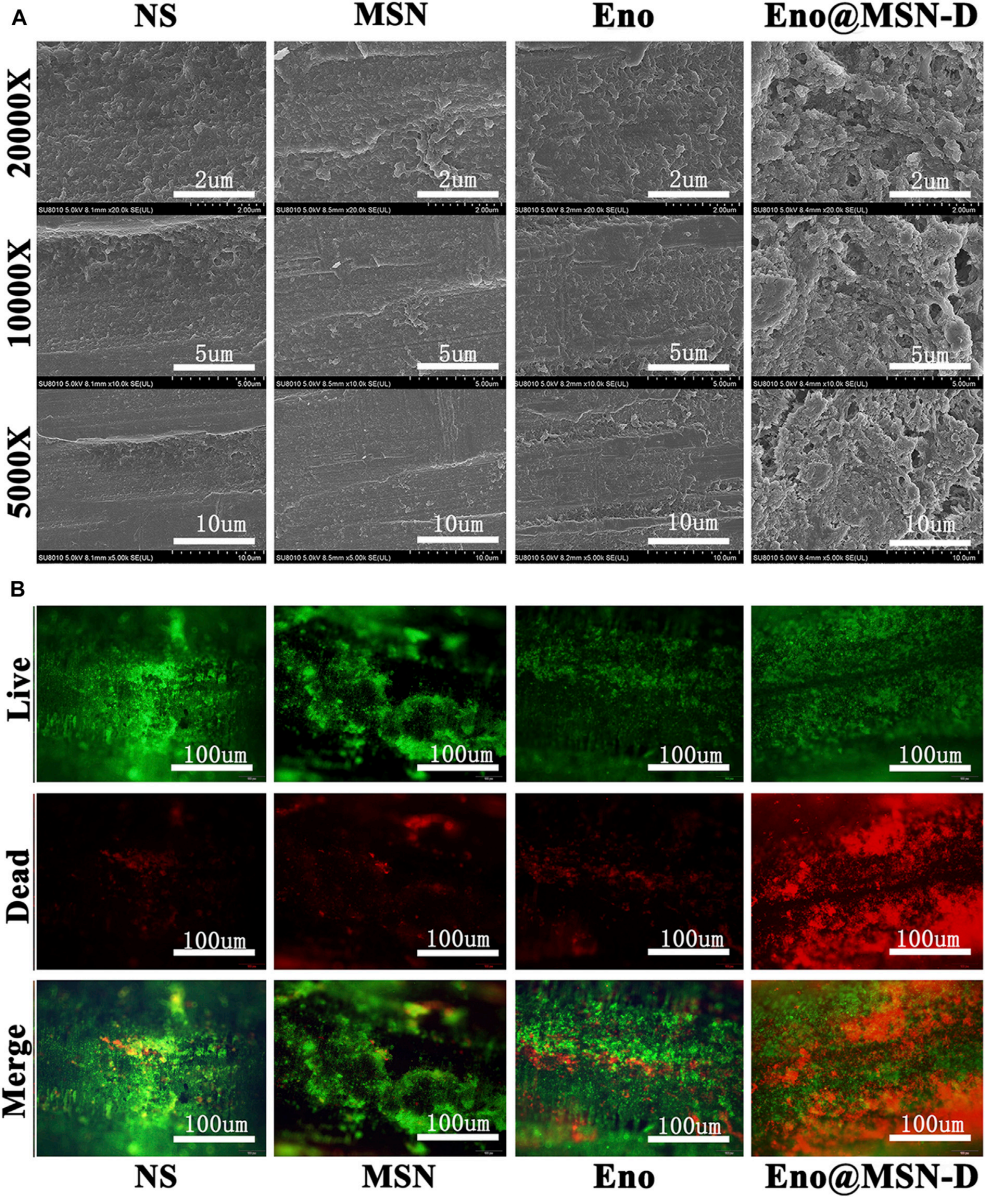
**(A)** SEM images of biofilm on the surface of the titanium rod. **(B)** The live dead bacterial staining of the biofilm on the surface of the titanium rod was observed under an inverted microscope.

Regarding the plate colony count of titanium rods after the ultrasound treatment, although colony number in the Eno@MSN-D group significantly differed from that in the Eno group, colony numbers between the MSN and NS groups was not significant (Figures 7C,D). The titanium rods were fixed with glutaraldehyde, dehydrated with ethanol, gold plated, and observed by SEM. As shown in Figure 8A, compared with other groups, including the NS, MSN, and Eno groups, the bacterial biofilm on the surface of the Eno@MSN-D injection titanium rod was loose, with large gaps in the biofilm. Furthermore, in the other groups, the titanium rod surface was not significantly different, and the biofilms on the surface of the other groups were more closely connected than that in the Eno@MSN-D group. We used the living/dead bacterial staining method to observe the bacterial biofilm on the titanium rod surface under an inverted fluorescence microscope. Figure 8B shows that the red fluorescence signal in the Eno@MSN-D group was more intense than that in the MSN, NS, and Eno groups. There was no significant difference in red fluorescence among the Eno, MSN, and NS groups, which shows that the Eno@MSN-D group was more substantial than other groups in terms of antibacterial performance *in vivo*.

### 3.7 Bone Morphometry Assay

**FIGURE 9:**
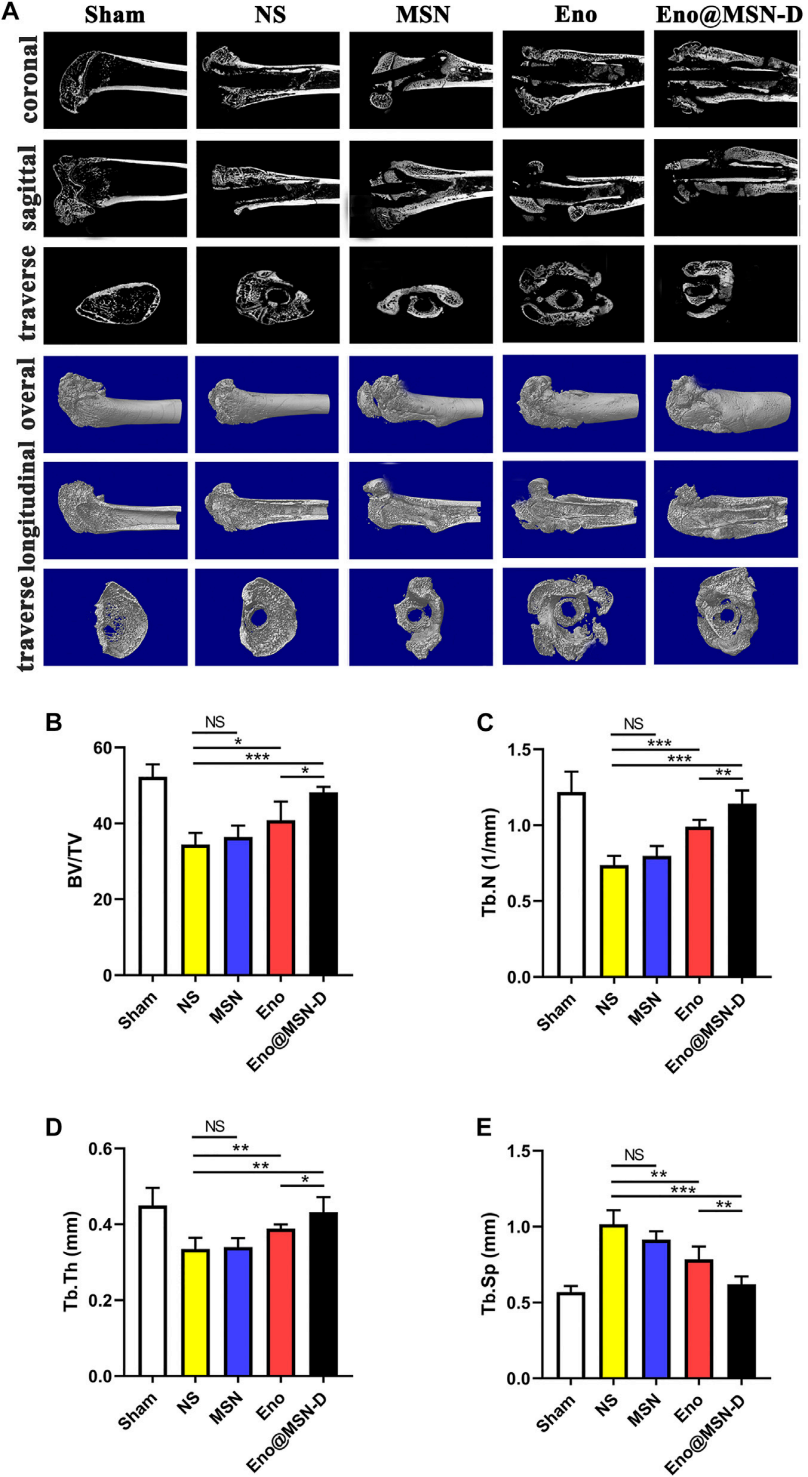
**(A)** Coronal, sagittal, and transverse two-dimensional micro-CT and integral, longitudinal, transverse three-dimensional micro-CT images of the femur. **(B–E)** Statistic diagram of bone volume fraction, number of bone trabeculae, trabecular bone thickness, and trabecular bone separation.

We used micro-CT to obtain two- and three-dimensional images from the femur. We also acquired coronal, sagittal, and transverse images of the two-dimensional images and the overall, longitudinal, and transverse three-dimensional images (Figure 9A). Compared with those in the Sham control group, the femur morphologies in the four experimental groups changed significantly, and osteolysis of different degrees was observed in all four groups. However, compared with the NS and MSN groups, there was evident bone formation around the titanium rod pores in the Eno@MSN-D and Eno groups. Besides, no significant difference was observed between the NS and MSN groups. By performing parameter analysis on the bone structure of the ROI and using the one-way variance analysis, we obtained some quantitative statistical graphs of the bone parameters (Figures 9B–E). The bone volume fraction (BV/TV), trabecular number (Tb.N), and trabecular thickness (Tb.Th) were higher in the Eno@MSN-D and Eno groups than in the NS group, and the Eno@MSN-D group is superior to the Eno group. However, trabecular bone separation (Tb.Sp) was considerably lower in both Eno@MSN-D and Eno groups than in the NS group. Based on the BV/TV, Tb.N, Tb.Th, and Tb. Sp, no noticeable difference was observed between the NS and MSN groups (*p* > 0.05). Overall, these results indicate that both Eno-@MSN-D and Eno groups effectively prevented osteolysis *in vivo.* Of note, the Eno-@MSN-D group was more effective than the Eno group.

### 3.8 Histopathology

**FIGURE 10:**
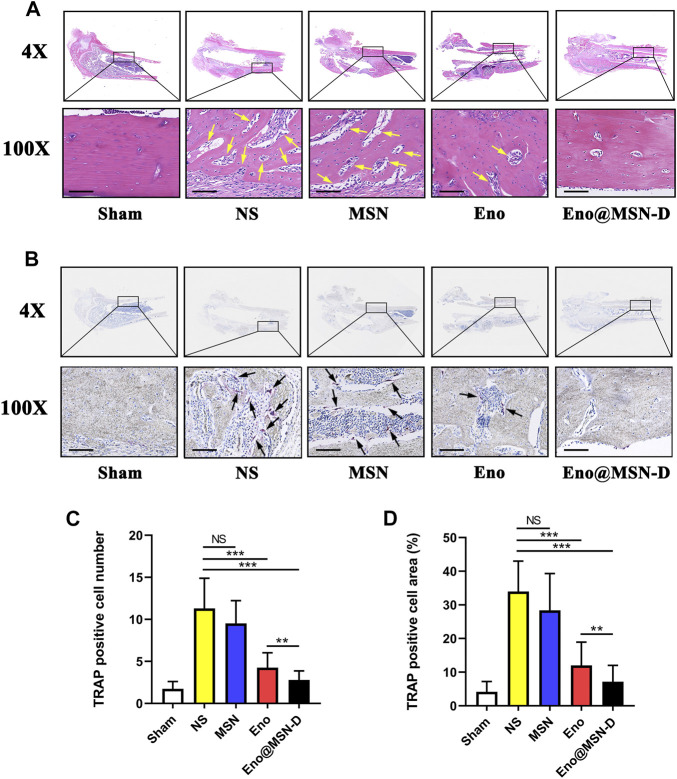
**(A)** The representative images of HE staining of the middle part of the femur. Yellow arrows indicate dimensional intracortical abscesses or inflammatory cells. **(B)** The representative images of TRAP staining of the middle part of the femur. Black arrows represent osteoclasts. **(C)** Statistical graph of the number of osteoclasts in each field of view. **(D)** Statistical graph of the area of osteoclasts in each field of view. All scale bars = 200 μm.

Figures 10A,B shows the longitudinal decalcified sections of different groups. The morphological changes in the left femur were detected by HE staining, and the osteoclasts around the pores of the titanium rod were detected by TRAP staining. Figure 10A shows more extensive bone cortex destruction and more abundant bone tissue death in the NS and MSN groups. Although there were signs of intramedullary inflammation in all four experimental groups, these were more severe in the MSN and NS groups. As shown in Figure 10B, the number of osteoclasts around the titanium rod pores was less in the Eno@MSN-D and Eno groups that in the NS group. Moreover, the number and area of osteoclasts in each group were measured, and quantitative analysis was performed (Figure 10C,D). Noteworthy, compared with the NS group, the number and area of osteoclasts in the Eno@MSN-D group and the ENO group were relatively fewer. Furthermore, the number and area of osteoclasts in the Eno@MSN-D group were lower than those in the ENO group.

## 4 Discussion

MSNs have been widely used in nanomedicine because they can be functionalised with chemical groups delivered to specific target sites to alter external organ organisation to accommodate specific target tissues (Chaudhary et al., 2019). However, despite these advances, challenges remain in effectively and sustainably controlling local bone drug release, calibrating the long-term stability and activity of drugs, while minimising cytotoxicity (Campoccia et al., 2010; Bagherifard, 2017). Regarding orthopaedic implant-related infections, nanoparticles are a promising strategy for overcoming biofilm formation and drug resistance (Russo et al., 2017).

In our study, we loaded enoxacin into MSN and coated the nanoparticles with PEG, which is phagocytised by macrophages. Simultaneously, bone-targeting D-Asp8 was connected to the nanoparticles to achieve targeted drug release in the bone tissue (Fu et al., 2014; Chen et al., 2018). Generally, bone adsorption molecules, such as bone-targeting ligands, are coupled to nano-carrier surfaces. The nano-carriers can chelate HA to achieve local drug deposition in bone tissue. After administration, a strong interaction between the bone-targeted ligands and HA leads to rapid retention and accumulation of the nano-carriers in the bone tissue. Therefore, this approach reduces drug leakage in the circulation and ensures highly localised antibiotic concentration (Swami et al., 2014). From the fluorescence signals in rats (Figure 6), it is evident that we successfully synthesised Eno@MSN-D nanoparticles that have excellent bone-targeting properties. Our data showed that the present MSNs had ultra-high specific surface areas and suitable pore sizes for enoxacin loading. During synthesis, the average particle size increased from MSN to Eno@MSN-D. The increase in particle size can be attributed to the loading of drugs inside or outside the pores of the monodisperse silica nanoparticles. The results are in accordance with those of a previous study (Wang et al., 2008), showing that the synthesised carrier size must be larger than that of the pure carrier material when the drugs and polymers are mixed with mesoporous silica nanoparticles.

The PDI reflects particle size uniformity, which is an important index for particle size characterisation. Within a specific distribution range, the smaller the distribution coefficient, the more uniform the particle size (Masarudin et al., 2015). Our data shows that the PDI of the synthesised Eno@MSN-D is the smallest compared with that of MSN and Eno@MSN, and therefore, it has a uniform particle size. The zeta potential of the carrier material can reflect the system’s stability, and it has an important influence on the release and circulation of body fluids. The carrier material stability in the surrounding fluid is an important index to overcome aggregate formation in the nano-drug delivery system (Wang et al., 2008). The higher the zeta potential (positive or negative) of the particles, the more stable the system is against aggregation (Yang et al., 2018). Our results show that the zeta potential of the synthesised Eno@MSN-D is −19.3 mVV, which is the smallest value; therefore, it has good dispersion properties. Besides, the TGA curves (Figure 2E) showed that the weight loss of Eno@MSN was higher than that of MSN, which indicated that enoxacin was loaded on MSN. In addition, the weight loss of Eno@MSN-D was higher than that of MSN and Eno@MSN; this was due to the loading of the enoxacin and the immobilisation of PEG and D-Asp8 on the surface of the nanoparticles. From the *in vitro* drug release curves of Eno@MSN-D in different pH response release patterns (Figure 2F), we determined two main causes of the phenomenon. Firstly, bacterial infections generally feature very low pH values due to their hypoxic nature (Benoit and Koo, 2016). The acidic environment of bacterial infectious sites can be harnessed in the design of pH sensitive drug delivery systems (Gupta et al., 2019). Moreover, *via* protonation at acidic pH, these properties result in greater drug release due to electrostatic repulsion within the nanocarriers (Radovic-Moreno et al., 2012; Zhu and Chen, 2015). These nanoparticles prevent non-specific interaction at physiological pH 7.4, thereby increasing the therapeutic activity with declining pH (Gupta et al., 2019). Secondly, it is due to the enoxacin molecular structure, as studies have reported that enoxacin is least water soluble at neutral pH and highest at acidic pH (Bedard and Bryan, 1989).

From the crystal violet staining, bacterial colony counting plate method unveiled the integrity of some bacterial morphology by SEM and live/dead bacterial staining, and a series of *in vitro* experiments showed that Eno@MSN-D has good antibacterial properties. From the treatment with Eno@MSN-D *in vivo*, it is apparent that the monodisperse silica microspheres were biocompatible with low toxicity, lacked immunogenicity, and degraded into non-toxic compounds (mainly silicic acid) in a short period (Lu et al., 2010). Additionally, our data show that Eno@MSN-D can inhibit osteoclast formation and function when the concentration is lower than the safe concentration of 20 μg/ml Eno@MSN-D.

The successful establishment of a rat infection model is the key to our confirmation that Eno@MSN-D has antibacterial properties *in vivo*. From SEM (Figure 8A), live/dead bacterial staining (Figure 8B), and bacterial colony plate count on the surface of femoral titanium rods (Figure 7C), we observed that Eno@MSN-D has antibacterial effect. In the quantitative bacterial colony plate count graphs (Figure 7D), the difference between the Eno@MSN-D and Eno groups as significant, which indirectly indicates that Eno@MSN-D had a better therapeutic effect. Similarly, we observed that, although there was no significant difference between the MSN and NS groups, the colony number in the MSN group was slightly lower; however, this was related to the antibacterial properties of the MSN. The inherent antibacterial activity of the nanoparticles was due to one of the three mechanisms: oxidative stress induction, metal ion release, or non-oxidative stress mechanism (Wang et al., 2017).

In the two- and three-dimensional rat femur images (Figure 9A), we observed the osteolysis phenomenon in the experimental group, which also confirmed the success of the rat bone infection model. Based on our data (Figures 9B–D), we found that the BV/TV, Tb.N, Tb.Th, and Tb. Sp were significantly different between the Eno@MSN-D and Eno groups, which indicated that Eno@MSN-D does play a role in preventing osteolysis *in vivo.* Of note, the Eno-@MSN-D group was more effective than the Eno group.

Bioactive silicon-based nanoparticles reportedly promote the differentiation and mineralisation of osteoblasts (Ha et al., 2014). Additionally, silicon ions released from MSNs have been shown to promote the formation of mineralised nodules, the synthesis of COL1, and the expression of osteogenic genes in osteoblasts (Sun et al., 2009). These observations suggest that MSN plays a role in osteogenesis, and thus may prevent osteolysis. Nevertheless, there was no significant difference in bone parameters between the MSN and NS groups, which indicates that the osteogenic capacity of MSN is still limited, and thus enoxacin still plays a significant role. Besides, the Eno@MSN-D had better osteogenesis abilities to prevent osteolysis, which is consistent with the histomorphological femoral HE (Figure 10A). The number and area of osteoclasts (Figures 10B–D) in the Eno@MSN-D and Eno groups were smaller than those in the NS groups. Furthermore, the Eno@MSN-D and EN groups significantly differed in terms of osteoclast number and area. Therefore, the Eno@MSN-D group could be more effective than the Eno group to prevent osteolysis.

The *in vitro* and *in vivo* studies of most nanoparticle-based sustained-release systems show that these materials show better antibacterial and antibiofilm activities, which is mainly related to reducing bacterial adhesion to grafts, higher effectiveness in drug-resistant strains, and/or controlling the antibiotic release. By obtaining sustained-release performance (Zhang et al., 2017; Liu et al., 2018; Zhang et al., 2019), we can achieve better local antibiotic concentrations and prolong the contact time between antibiotics and bacteria, which is essential for removing biofilms. Additionally, carriers offer advantages in terms of therapeutic drug release kinetics, ease of manufacture, biodegradability, chemical binding relative to physical binding, and excellent transport characteristics. Thus, these biomaterials will play a more critical role in medical diagnosis and treatment processes.

## 5 Conclusion

Bacterial colonisation and biofilm formation are issues in post-implantation infections with *S. aureus*. Although conventional antibiotics limit the formation of bacterial biofilms, they ignore the fact that bacterial biofilms restrict the spread of antibiotics to the infected area, further lowering the concentration of antibiotics available to the infected area, as well as bone loss caused by osteoclast formation following orthopaedic implant-associated infections. Despite the dual antibacterial and osteoclast-inhibiting effects of enoxacin, it has limited penetration into bone tissue to achieve adequate blood levels. Fortunately, MSN has been used for the intracellular delivery of antibiotic targets against intracellular infections and bone loss over the last few decades. In this study, we successfully prepared and characterised Eno@MSN-D nanoparticles-based on MSN. Our data show that the synthesised Eno@MSN-D is a safe drug delivery system that enables sustained-release of specific antibiotics to target *S.* aureus-related post-operative implantation infections. Eno@MSN-D not only has excellent antibacterial properties and osteoclast inhibitory properties *in vitro* but also allows the specific targeting of bone tissues to prevent bone loss *in vivo*. Therefore, this method provides a new way to treat and prevent post-operative, orthopaedic *S. aureus*-related implantation infections and bone loss.

## Data Availability

The original contributions presented in the study are included in the article/Supplementary Material, further inquiries can be directed to the corresponding authors.
